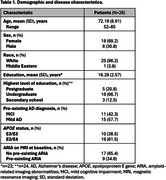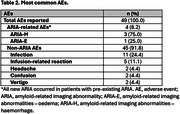# Lecanemab in the real world: First UK experience in the new era of disease modifying treatments for Alzheimer’s disease

**DOI:** 10.1002/alz70861_108515

**Published:** 2025-12-23

**Authors:** Avinash Kumar Hari Narayanan, Emer MacSweeney

**Affiliations:** ^1^ Re:Cognition Health, London, Greater London UK; ^2^ Re‐Cognition Health, London, Greater London UK

## Abstract

**Background:**

Lecanemab is an anti‐amyloid monoclonal antibody demonstrated to slow cognitive decline in early Alzheimer’s disease (AD) in clinical trials. In August 2024, it was approved in the UK for the treatment of AD patients with mild cognitive impairment or early dementia who are apolipoprotein E4 heterozygotes or non‐carriers. Here, we present a clinical snapshot of the first 26 patients treated with lecanemab in a real‐world UK setting. Our findings offer early insights into the novel use of anti‐amyloid therapy in clinical practice, marking a new era in AD treatment.

**Method:**

Patients referred to Re:Cognition Health, London, underwent screening to confirm AD diagnosis and eligibility for lecanemab. Lecanemab infusions commenced in November 2024. Collected data includes patient demographics, disease and treatment characteristics, and adverse events (AE). Patient experience was evaluated using the standardised Quality of Life in Alzheimer's Disease (Qol‐AD) patient/caregiver survey.

**Result:**

41 AD patients were referred for anti‐amyloid therapy, of whom 26 consented and were eligible to receive lecanemab. The mean cohort age was 72 years with 69% patients being female (Table 1). Most patients (81%) had university‐level education. All patients had confirmed amyloid biomarker status, verified during or prior to screening via neuroimaging or lumbar puncture. The mean (standard deviation) time from diagnosis to first infusion was 26.2 (22.4) months, while screening to infusion time was 1.3 (1.3) months. A total of 49 AEs were reported in 20 patients (76.9%), with non‐amyloid‐related imaging abnormalities (ARIA) AEs comprising 91.8% of all events (Table 2). The most frequently reported AEs included infection (24.4%), infusion‐related reactions (11.1%), headache (4.4%), confusion (4.4%) and vertigo (4.4%). ARIA was observed in 4 patients (8.2%), all of whom had pre‐existing ARIA at baseline. No serious AEs were reported. Results from the ongoing QoL‐AD survey show that patients felt better since initiating lecanemab treatment.

**Conclusion:**

This report presents the first real‐world experience of lecanemab treatment for AD patients in the UK. Our findings demonstrate feasibility of anti‐amyloid therapy in clinical practice, manageable safety profile and early indications of positive patient‐reported outcomes. These insights can inform service development and support clinical uptake of novel disease‐modifying therapies.